# Genome-wide phylodynamic approach reveals the epidemic dynamics of the main *Mycoplasma bovis* subtype circulating in France

**DOI:** 10.1099/mgen.0.001067

**Published:** 2023-07-24

**Authors:** Julien Thézé, Chloé Ambroset, Séverine Barry, Sébastien Masseglia, Adélie Colin, Agnès Tricot, Florence Tardy, Xavier Bailly

**Affiliations:** ^1^​ Université Clermont Auvergne, INRAE, VetAgro Sup, UMR EPIA, Saint-Genès-Champanelle, France; ^2^​ Université de Lyon, ANSES, VetAgro Sup, UMR Mycoplasmoses animales, Lyon, France

**Keywords:** surveillance, lineage replacement, fitness, respiratory disease, cattle, bacteria

## Abstract

*
Mycoplasma bovis* is a major aetiological agent of bovine respiratory disease worldwide. Genome-based analyses are increasingly being used to monitor the genetic diversity and global distribution of *M. bovis *, complementing existing subtyping schemes based on locus sequencing. However, these analyses have so far provided limited information on the spatiotemporal and population dynamics of circulating subtypes. Here we applied a genome-wide phylodynamic approach to explore the epidemic dynamics of 88 French *M. bovis* strains collected between 2000 and 2019 in France and belonging to the currently dominant *polC* subtype 2 (st2). A strong molecular clock signal detected in the genomic data enabled robust phylodynamic inferences, which estimated that the *M. bovis* st2 population in France is composed of two lineages that successively emerged from independent introductions of international strains. The first lineage appeared around 2000 and supplanted the previously established antimicrobial-susceptible *polC* subtype 1. The second lineage, which is likely more transmissible, progressively replaced the first *M. bovis* st2 lineage population from 2005 onward and became predominant after 2010. Analyses also showed a brief decline in this second *M. bovis* st2 lineage population in around 2011, possibly due to the challenge from the concurrent emergence of *M. bovis polC* subtype 3 in France. Finally, we identified non-synonymous mutations in genes associated with lineages, which raises prospects for identifying new surveillance molecular markers. A genome-wide phylodynamic approach provides valuable resources for monitoring the evolution and epidemic dynamics of circulating *M. bovis* subtypes, and may prove critical for developing more effective surveillance systems and disease control strategies.

## Data Summary

The authors confirm that all supporting data, code and protocols have been provided within the article or through supplementary data files.

**Impact Statement**

Bovine respiratory disease is the most common cause of morbidity and mortality in young cattle worldwide. One of the bacteria most consistently implicated in this complex, multifactorial disease is *Mycoplasma bovis *. Genome-based studies are increasingly adopted to capture and monitor the genetic diversity and global distribution of *M. bovis *, but have so far provided limited information on *M. bovis* epidemic dynamics. Due to inherent methodological limitations, bacteria have so far been less investigated by phylodynamic approaches than RNA viruses. Here we used a genome-wide phylodynamic approach to gain insight into the spatiotemporal and population dynamics of *M. bovis polC* subtype 2 (st2), the main subtype circulating in France over the last two decades. Our results show the successive emergence and nationwide dissemination of at least two introduced *M. bovis* st2 genotypes. Our results also underline central role of introductions, notably of fitter and potentially more transmissible genotypes that can rapidly supplant pre-existing *M. bovis* populations. This work highlights the need for more consistent genome-based surveillance and for greater adoption and development of phylodynamic methods, as they could transform the way surveillance systems and disease control measures are designed for animal health.

## Introduction

Since their introduction in the early 2000s, phylodynamic methods have increasingly been used to unravel the evolutionary and epidemic dynamics of pathogen populations [1]. They have been used extensively for fast-evolving organisms, in particular human RNA viruses, as they can identify nucleotide variations in genomes that serve to estimate pathogen evolution and transmission dynamics over the course of a single epidemic [2, 3]. A recent review highlighted the value of phylodynamic approaches to help understand patterns of disease transmission and design more effective control strategies in animal health research [4]. Phylodynamic methods can also be employed to monitor outbreaks caused by bacteria or other slow-evolving pathogens, although with some limitations [5]. In particular, regular longitudinal sampling over a long period of time is usually required to counterbalance the poor molecular clock signal contained in the genetic data [6]. Analyses also have to contend with potentially high recombination rates that could have a significant impact on genome-wide phylogenetic reconstructions [7].

The genus *Mycoplasma* comprises more than 200 species that are pathogens, opportunists or commensals of a wide range of animal hosts, including humans. *Mycoplasma* characteristically lack a cell wall and have small genomes (ranging from 0.58 to 1.84 Mb). In the tree of life, *Mycoplasma* spp. appear in the longest branches, suggesting fast genome evolution [8]. A genome-wide study estimated a nucleotide substitution rate of 0.8–1.2×10^−5^ per site per year for the bird and poultry pathogen *Mycoplasma gallisepticum* across a 13 year sampling [9]. This estimate counts among the highest reported for a bacterium [10], and holds promise for applying phylodynamic approaches to *M. gallisepticum* and other *Mycoplasma* species. Phylodynamic approaches have recently been assessed in an effort to explore the origin and spread of *M. gallisepticum* in Ecuador, but the study remains limited as the phylodynamic inferences were based on the sequence of a single gene [11].

In ruminants, another mycoplasma, *Mycoplasma bovis *, is a major cattle pathogen that causes huge economic and productivity losses worldwide. Clinical *M. bovis *-related infections mainly include respiratory diseases in young cattle and mastitis and arthritis in adults [12]. *M. bovis* is endemic in a large number of countries. As with many farm animal infections, surveillance is non-mandatory and often passive, relying on centralized data and strains provided from pathogen detection assays to support in-field diagnosis [13, 14]. Genetic typing approaches have been proposed as a way to monitor potential emergences worldwide [15] or at national levels [16, 17]. The recent use of whole-genome sequencing has also opened up new avenues for studying strain diversity and relatedness worldwide [18–21], and has already shown that *M. bovis* strains cluster according to their geographical origin and potentially disseminate worldwide through cattle trade [21].

France has a long history of *M. bovis* infections. French epidemiological studies based on *polC* gene single-locus sequence typing have demonstrated the dominant spread in the early 2000s of a clonal *M. bovis* population (subtype 2 [st2]; ST8 in the multilocus sequence typing [MLST] scheme developpped by Register et *al.* [15]) with acquired resistance to most antimicrobial families except fluoroquinolones [16, 22]. The emergence of st2 was concomitant with the decline of the previously established subtype 1 (st1) in France [16]. Another subtype named st3 was detected for the first time in 2011 and currently accounts for up to 20 % of the circulating *M. bovis* population in France [18, 23]. A recent study examined the genomic features of each of the three *M. bovis* subtypes circulating in France in detail using one prototype strain per subtype [24] and found a relatively balanced profile for *M. bovis* st2 in terms of mobile genetic elements, which possibly explains how it became the predominant subtype circulating in France. However, such data are still of limited use for defining the burden and dynamics of circulating *M. bovis* populations or for determining the precise impact of introductions.

In this study, we investigated the ability of genomic epidemiology to gain insights into the emergence and spread of the *M. bovis* st2 lineage in France. We sequenced the genomes of a large set of representative *M. bovis* st2 strains collected in France since the date this subtype was first detected. We applied phylodynamic approaches on genome data to identify the circulation of two *M. bovis* st2 lineages and to investigate their respective introductions and population dynamics. Finally, we determined new sets of molecular markers that could prove useful for *M. bovis* surveillance.

## Methods

### Sample collection, isolation and subtyping

**Fig. 1.**
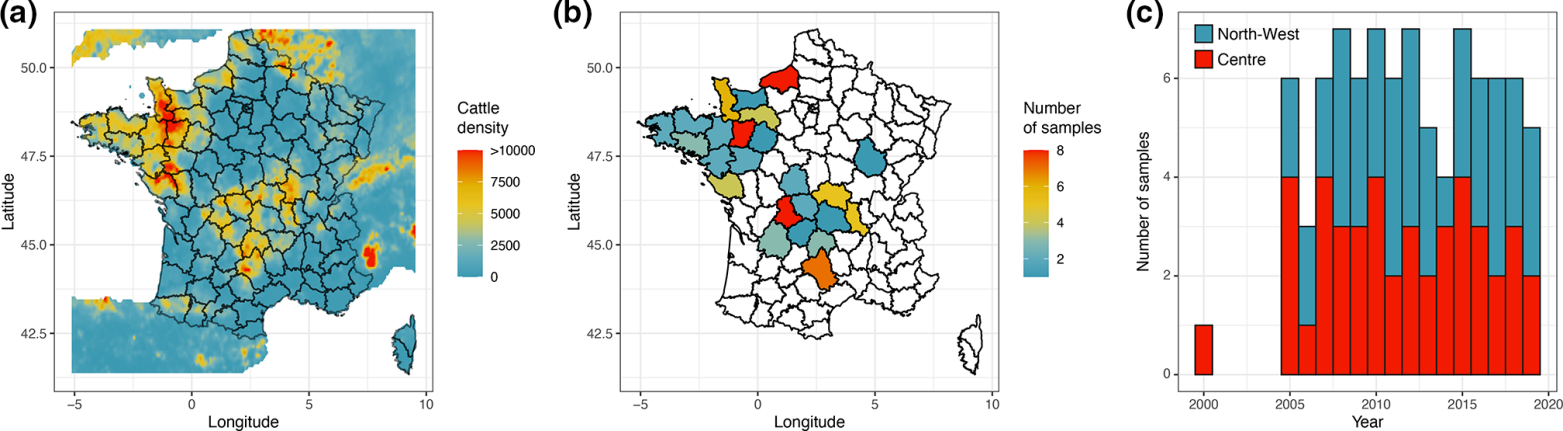
Geographical and temporal distribution of *Mycoplasma bovis* samples from this study and cattle density. (**a**) Map of France showing department sub-divisions. The colour gradient indicates cattle density km^−^² extracted from Gilbert *et al*. [61]. (**b, c**) Sampling location and temporal distribution of *M. bovis* isolates from the two most cattle-populous regions of France, i.e. North-West and Centre.

*M. bovis* isolates and associated metadata were collected in the framework of the Vigimyc passive epidemiological surveillance network [14]. The isolates originate from calves with respiratory disorders sampled in France between 2000 and 2019, corresponding to the known circulation of *M. bovis* st2 (Fig. 1 and Table S1, available in the online version of this article). As we had little prior knowledge of *M. bovis* st2 epidemiology, we performed a geographical and temporal uniform sampling that should be appropriate for phylodynamic analyses [25]. We selected an equivalent number of isolates from the two most cattle-populous regions of France, i.e. North-West and Centre (Fig. 1a, b and Table S1), which we assumed to be the main drivers of *M. bovis* circulation. We also selected a similar number of isolates per year from each of these regions when possible (Fig. 1c and Table S1).

Isolates were grown for ~30 h at 37 °C under 5 % CO_2_ in pleuro-pneumonia-like organism broth supplemented as previously described [26]. Genomic DNA was extracted from 4 ml cultures of each strain using a commercial kit from Lucigen (Epicentre). The quality of the extracted DNA was checked using a Nanodrop spectrophotometer (Thermo Fisher) using the optical densities OD_260/280_≥1.8 and OD_260/230_≥ 2. The concentration of the extracted DNA was checked using a Qubit fluorimeter (dsDNA BR assay kit, Thermo Fisher). A total of 88 isolates belonging to *M. bovis* st2 as determined by polC gene sequencing [16] were selected for genome production (Fig. 1 and Table S1).

### Genome sequencing, assembly and annotation

Sequencing libraries were prepared using the Illumina Nextera XT library preparation kit and multiplexing index set A, with an input of 0.2 ng µl^−1^ of genomic DNA. We followed the recommended library preparation workflow from Illumina, with one small change regarding the beads used for the clean-up step: we used Macherey-Nagel beads (NUCLEOMAG NGS Clean-up and Size Select #744970.5) and not AMPure XP beads as recommended in the standard Illumina protocol. The quality and average size of the libraries were checked using an Agilent 2100 Bioanalyzer by migrating 1 µl of each library onto high-sensitivity DNA chips. Each library was quantified in QubitFlex with a Qubit dsDNA HS assay kit, normalized to 1 nM (based on the average size obtained in the Bioanalyzer) and then mixed to obtain a pool of 96 libraries at 1 nM. Sequencing was performed on an Illumina iSeq100 benchtop sequencer using 20 µl of the pool diluted to 100 pM (paired-end runs and 151 cycles). The sequencing was considered robust and consequently stopped when mean coverage depth was above 30×. Mean coverage depth was evaluated by mapping reads on the historical reference strain PG45^T^ (accession number: NC_014760) [27] using bwa mem [28].

Raw reads were demultiplexed, followed by a deduplication step using SuperDeduper [29] and then quality trimming based on fastQC results [30]. Filtered reads were assembled using SPAdes 3.15.1 [31] with the default parameters to obtain *de novo* draft assemblies. Protein coding sequences (CDSs) were annotated on the resulting scaffolds using Prokka v1.14.5 [32] with the *Mycoplasma* genetic code.

### Genomic data collation and core-genome alignments

We collated all the genomic data publicly available from the National Center for Biotechnology Information (NCBI, last accessed July 2022), including 138 complete and near-complete genomes (including two French *M. bovis* st2 complete genomes, strain L15762 [24] and strain RM16 [33]), 373 assembled draft genomes (whole-genome shotgun; WGS) and 950 raw read datasets (Short-Read Archive; SRA). For each category, we retrieved, when available, the isolate name, country of origin and date of collection. *De novo* draft assemblies were obtained from raw read datasets (SRA) using SPAdes. NCBI-based complete and draft genomes (including WGS and SRA datasets) were subsequently annotated using Prokka, as described above.

The Panaroo 1.2.10 pangenome investigation pipeline [34] was used to generate two core-genome alignments, one including only the French *M. bovis* st2 isolates of this study (called French *M. bovis* st2 core-genome) and a second adding a reduced public dataset of 339 *M *. *bovis* isolates (called *M. bovis* complete core-genome). This latter dataset corresponds to the NCBI WGS and SRA datasets that we subsampled to reduce genetic and location redundancy, while also deliberately removing all Belgian complete genomes from the study by Bokma *et al*. [19] due to the numerous frameshifts in CDSs. Finally, an in-house script was applied to core-genome alignments to retain only one gene copy for paralogues and keep only sequences in gene alignments with a length equal to the most common length to discard disrupted CDSs. The two core-genome alignments thus obtained only contained orthologous genes present in one copy in 95 % of the taxa.

### Phylogenetic and molecular clock analyses

The full core-genome alignments were used to reconstruct maximum-likelihood (ML) phylogenies using IQTREE v2.0.3 [35] and the general time-reversible nucleotide substitution model, a gamma distribution and a proportion of invariable sites (GTR+G+I), as estimated by ModelFinder [36], bundled into IQTREE. Statistical support for nodes of the ML phylogenies was assessed using an ultrafast bootstrap approach of 1000 replicates implemented in IQTREE. A consistency index (CI), using the Phangorn R package [37] was calculated between the French *M. bovis* st2 ML phylogeny and parsimony-informative sites of the *M. bovis st2* core-genome alignment. The consistency index measures the level of homoplasy in sequence data and is equal to one if there is no homoplasy.

A root-to-tip analysis using the root-to-tip function of the BactDating R package [38] was performed on the French *M. bovis* st2 ML phylogeny to measure the molecular clock signal contained in the genomic data. A time-scaled Bayesian phylogeny was estimated on the full French *M. bovis* st2 core-genome alignment using beast v1.10.4 [39]. Bayesian analyses were computed with two independent runs of 50 million Markov chain Monte Carlo (MCMC) steps, sampling parameters and trees at every 5000 steps. We first conducted molecular clock (strict or relaxed clock) and demographic (constant population size, exponential growth or a SkyGrid coalescent model [40] that can estimate complex demographic trajectories) model comparisons using path sampling and stepping-stone marginal-likelihood estimators [41] to select the most appropriate priors for our dataset. Based on the resulting best log marginalized likelihoods (data not shown), we used the substitution model and parameters GTR+G+I, an uncorrelated lognormal relaxed clock [42] with a non-informative continuous-time Markov chain (CTMC) reference prior [43] placed on the molecular clock rate, and a SkyGrid coalescent demographic model [40].

The Tracer v1.7 program [44] was used to evaluate MCMC chain convergence and compute marginal posterior distributions of parameters, after first removing 10 % of the chain as burn-in. Logcombiner was used to combine and subsample posterior distributions, after removing 10 % of the chains as burn-in. TreeAnnotator was used to generate a summary maximum clade credibility (MCC) tree from the posterior distribution of trees. Phylogenies were drawn using the ggtree package [45] of the R software platform (http://www.R-project.org/)

### *In silico* fitness analyses

An analysis was performed to estimate relative fitness [46] variation in the French *M. bovis* st2 ML tree. This approach [47] uses branching patterns of phylogenetic trees to extract information about the relative fitness of an individual compared to others in a phylogeny. It assumes that evolution proceeds by progressive accumulation of small-effect mutations, and can be applied to any asexual population under persistent selection pressure.

### Birth–death skyline analyses

We estimated the effective reproductive number (*R*_e_) for two sub-datasets of the full French *M. bovis* st2 core-genome alignment using the serially sampled birth–death skyline model [48] implemented in beast 2 v2.6.6 [49]. We computed two independent runs of 100 million MCMC steps and sampled parameters every 5000 steps. In each run, as in the molecular clock analysis above, we used the substitution model and parameters GTR+G+I, and an uncorrelated lognormal relaxed clock model [42] with an uninformative uniform prior placed on the molecular clock rate parameter. A lognormal prior with a mean of 0 and a standard deviation of 1.00 was placed on the *R*_e_ and the becoming-uninfectious rate parameters. A beta prior with parameters a and b both set to 1 was placed on the sampling proportion, and a uniform prior was placed on the origin time and bounded to be no older than 1990. *R*_e_ and sampling proportion parameters were allowed to change at 15 time points equally spaced between the time to most recent common ancestor (TMRCA) and the time of the most recent sample.

Tracer v1.7 was used to check MCMC chain convergence, and logcombiner was used to combine and subsample posterior distributions after removing 10 % of the chains as burn-in. Figures were produced in the R software platform using in-house scripts and the R package bdskytools (available at https://github.com/laduplessis/bdskytools).

## Results

### Sample collection and genomic characteristics

A total of 88 French *M. bovis* st2 isolates collected from calves with respiratory disease were included in the study. These isolates originated in equal proportions from the two most cattle-populous regions of France, i.e. 44 samples isolated from each of the North-West and Centre regions (Fig. 1a, b and Table S1). The Vigimyc surveillance network provides exceptionally good temporal sampling ranging over almost 20 years. We were able to retrieve three to seven samples per year from 2005 to 2019 (Fig. 1c and Table S1) plus one sample isolated in 2000 (strain no. 2357). The geographical distribution of samples is maintained throughout the studied period with a median of three samples per year for both the North-West and Centre regions, expect for 2000 (Fig. 1c and Table S1).

Isolates were subsequently sequenced and *de novo* assembled to obtain scaffolds with a median N50 of 11 654 bp, a median genome size of 950 174 bp and a median predicted CDS number of 775 (Table S1). These characteristics were consistent with general genomic features (size and number of CDSs) of *M. bovis* genomes retrieved in public databases.

### Genetic diversity of *M *. *bovis* in France

A pan-genome analysis on the complete *M. bovis* dataset (88 French *M. bovis* st2 isolates sequenced in this study and 339 subsampled public *M. bovis* sequences) identified 613 core genes and 509 accessory genes. After a curation step to remove paralogues and disrupted CDSs, we obtained a core-genome alignment that includes 346 genes and has a total length of 320 826 nucleotides. The ML phylogenetic tree based on the complete *M. bovis* core-genome alignment (Fig. S1) shows several large and well-supported monophyletic lineages, consistent with the *polC* gene fragment subtyping, showing three well-supported monophyletic groups corresponding to subtypes st1 and st2 and strains with a *polC* sequence identical to HB0801 [50], grouped as st4. It also shows one well-supported paraphyletic group corresponding to st3 that includes the st4 clade (Fig. S1). This phylogeny demonstrates the robustness of the *polC*-based subtyping genetic marker, as only 24 out of the 427 isolates (5.6 %) diverged between the *M. bovis* core-genome phylogeny and their *polC-*subtype.

French *M. bovis* st2 isolates (88 isolates sequenced here and 9 previously sequenced) fall into one monophyletic group, with the exception of isolate 8149 that falls into another group including Israeli and European isolates (Fig. S1). Given that only one French isolate belongs to this group, we set aside isolate 8149 and focused all further analyses on the main *M. bovis* st2 clade (Fig. S2), which includes 122 isolates (i.e. 96 French, 2 Israeli, 5 Lithuanian, 3 Hungarian and 15 Spanish). The Israeli, Lithuanian and Hungarian isolates were connected to internal nodes with long branches, while the Spanish isolates were distributed among French isolates with short branches.

### Temporal variation in relative fitness

A second pan-genome analysis was performed on the 87 French *M. bovis* st2 isolates of this study belonging to the dominant lineage plus the L15762 isolate considered to be the type strain for French *M. bovis* st2 isolates [24]. The resulting pan-genome comprised 891 CDSs, including 656 core genes and 235 accessory genes. After curation of paralogues and disrupted CDSs, we obtained a French *M. bovis* st2 core-genome alignment with 531 genes and a total length of 501 516 nucleotides. It should be noted that only 147 out of the 830 polymorphic sites were parsimony-informative and those show a consistency index of 0.88 with *M. bovis* st2 core-genome phylogeny, indicating that the *M. bovis* st2 core-genome alignment contained few homoplasic sites.

**Fig. 2.**
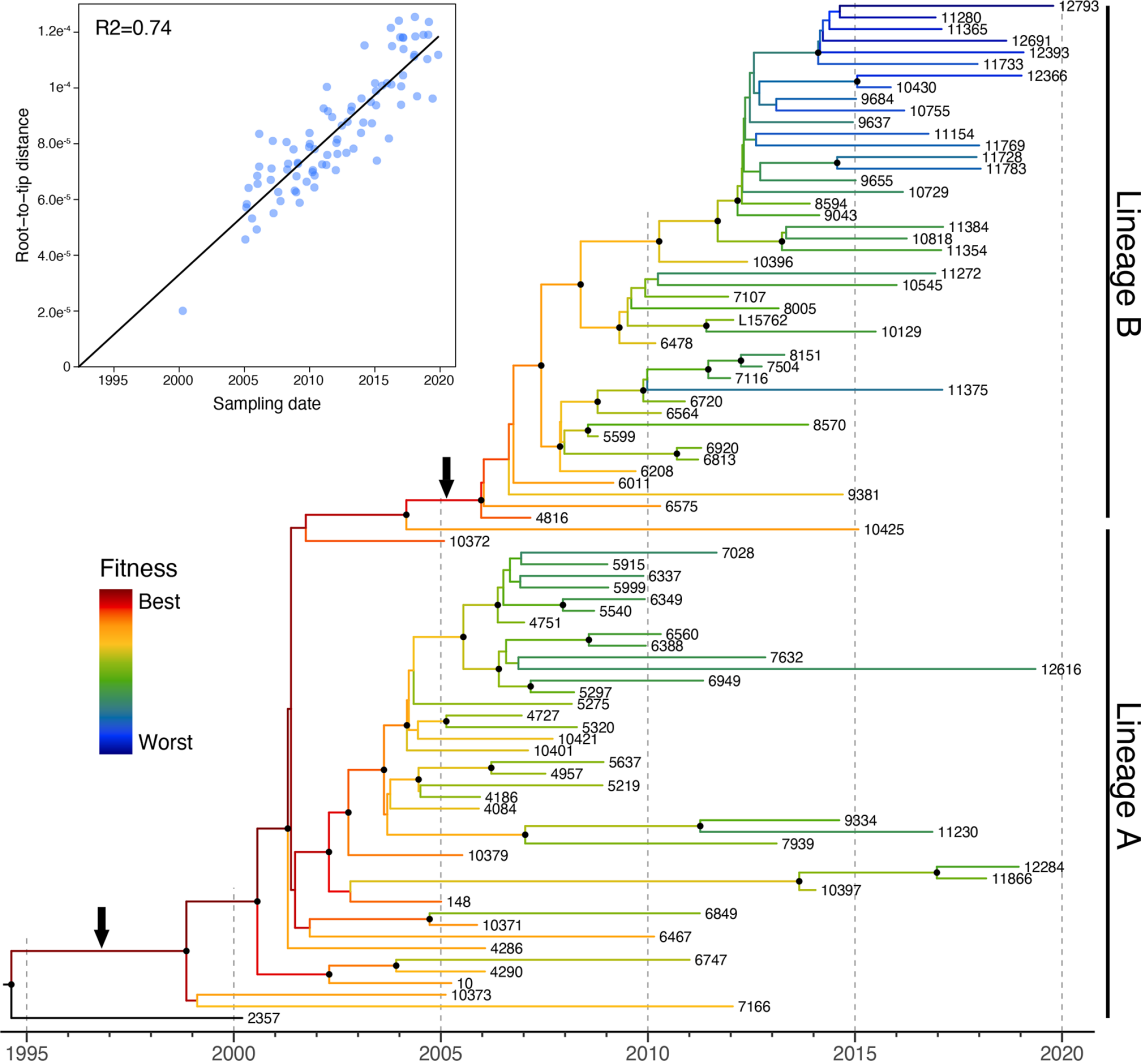
Fitness-annotated time-scaled phylogeny of *Mycoplasma bovis* subtype 2 in France. A maximum-credibility clade (MCC) estimated from a Bayesian molecular clock analysis using the core-genome alignment of 88 French *M. bovis* subtype 2 isolates (87 genomes generated in this study and the L15762 reference genome). The phylogeny is temporally rooted. Branch colours indicate relative fitness between lineages in the phylogeny. Black circles at internal nodes denote posterior clade probabilities >0.75. Black arrows indicate internal branch links with samples outside France (Fig. S2). The top-left panel shows the regression analysis between the sampling dates and the root-to-tip distances in the maximum-likelihood phylogenetic tree of 88 French subtype 2 *M. bovis* isolates (see Fig. S3).

The ML phylogenetic tree based on the French *M. bovis* st2 core-genome alignment (Fig. S3) showed no spatial structuration per French departments, regions or production areas (non-significant association index; data not shown). In contrast, it exhibited a ladder-like shape with high variations in branching rate and the more recent the samples, the more distant they were from the root, suggesting strong population dynamics and a strong temporal structure of the population (Fig. S3). We therefore performed both a molecular clock analysis and a fitness analysis. A root-to-tip analysis showed a strong molecular clock signal within our dataset, with a significant correlation of R^2^=0.74 (Fig. 2, Fig. S3). This allowed a Bayesian molecular clock analysis to be conducted confidently. The analysis estimated a mean clock rate of 4.86×10^−6^ substitutions per site per year. The resulting time-scaled phylogeny of the French *M. bovis* st2 isolates of this study (Fig. 2) still showed a ladder-like shape but with several changes in branching rates, indicating important variations in population dynamics over time. In this regard, the phylogeny showed two distinct and well-supported groups of related *M. bovis* st2 isolates (called here lineages A and B, Fig. 2) that differ in terms of temporal distribution. The paraphyletic lineage A accounted for most of isolates between 2000 and 2010, while the derived monophyletic lineage B comprised most of the isolates between 2010 and 2019. The fitness analysis performed on the tree using the approach described by Neher *et al.* [44] singled out some internal branches that had a better relative fitness compared to contemporaneous lineages in the phylogenetic tree, notably the one leading to the root of lineage B (Fig. 2). This suggests that lineage B with a higher relative fitness has replaced lineage A.

Interestingly, both lineage A and lineage B shared most recent common ancestors with samples isolated outside France, in Europe (Lithuania or Hungary) or Israel (Figs 2 and S2), suggesting that they emerged from independent introductions of international *M. bovis* st2 strains into France. Lineage A was a first successful *M. bovis* st2 introduction into France in the late 1990s that replaced the initial French *M. bovis* st1 population. Lineage B was introduced into France later on, in around 2005, and replaced the established lineage A population. Another *M. bovis* st2 introduction into France was detected in the late 1990s, represented by strain 2357 isolated in 2000. Our sampling after 2005 did not reveal any other strains belonging to this lineage, suggesting that it did not result in a large, long-lasting epidemic in France.

### Population and epidemic dynamics

**Fig. 3.**
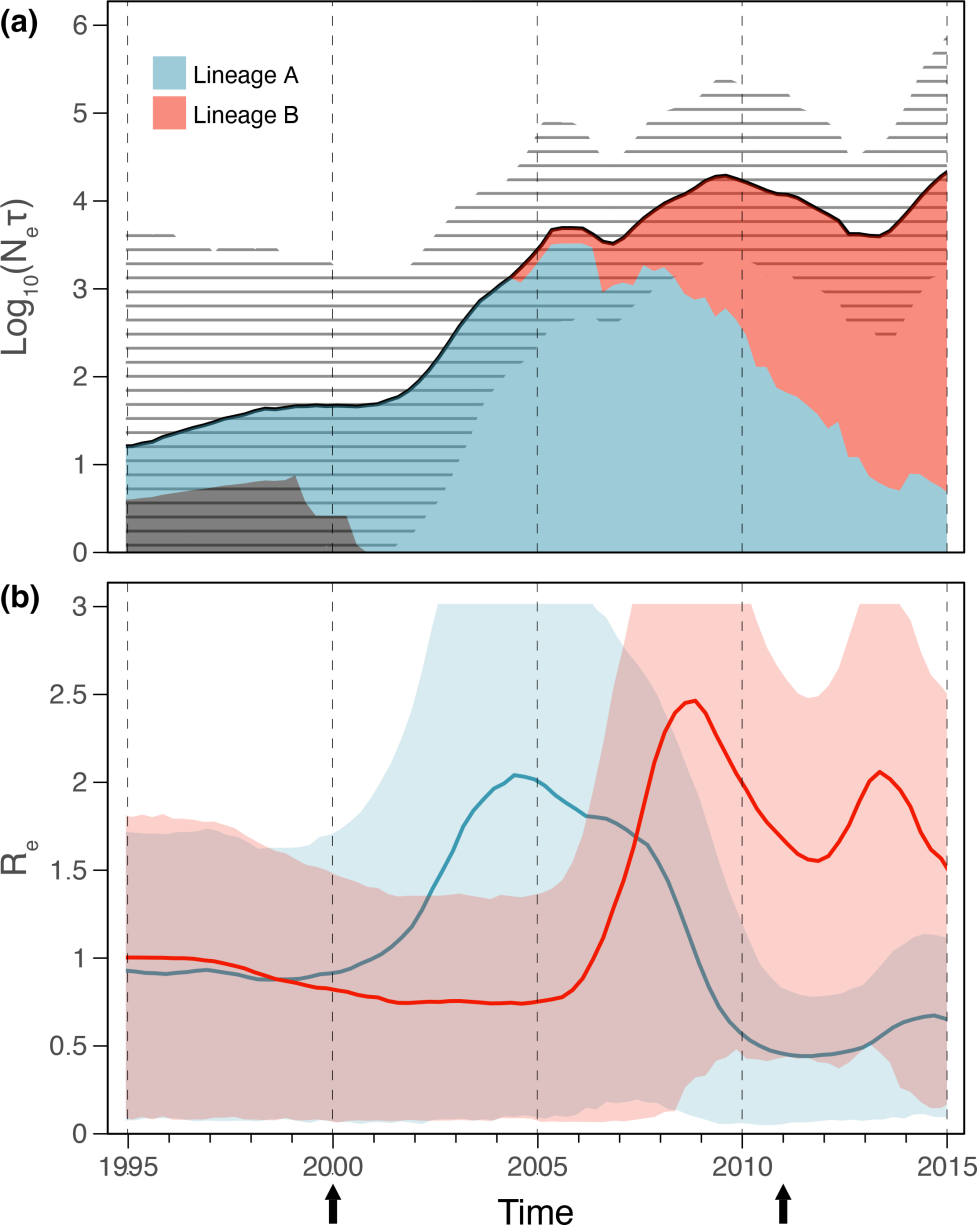
Population dynamics of *Mycoplasma bovis* subtype 2 in France. (**a**) Effective population size (*N*_e_) through time, estimated using a Bayesian SkyGrid approach. The black line and grey hatched area represent the posterior median estimate of *N*_e_ and its 95 % posterior highest credible density intervals, respectively. Blue and red areas indicate the relative *N*_e_ proportion of lineages A and B, estimated using lineage-through-time analysis. (**b**) Effective reproductive number (*R*_e_) through time, estimated using a birth–death skyline approach. The blue and red lines and shadings represent posterior median *R*_e_ estimates of lineages A and B and their 75 % posterior highest credible density intervals, respectively. Black arrows indicate the first detection of *M. bovis* subtypes 2 (in 2000) and 3 (in 2011) in France through the Vigimyc epidemiological surveillance network.

We used a Bayesian coalescent SkyGrid approach combined with a lineage-through-time measure to estimate the effective population size (*N*_e_) of the French *M. bovis* st2 population and the relative contribution to the overall population of lineages A and B (Fig. 3a). We observed a first period of steady population growth from 2002 to 2005 corresponding to the introduction and subsequent establishment of lineage A in France in the late 1990s that was first detected in 2000 through the French Vigimyc surveillance system. This was followed by a slight decline in 2006 occurring concurrently with the introduction of lineage B that progressively came to dominate the French *M. bovis* st2 population. We observed a second period of decline from 2010 to 2013 concurrently with the first detection and isolation in 2011 of *M. bovis* st3 isolates in mainland France.

We used a Bayesian birth–death skyline approach to estimate the effective reproductive number (*R*_e_) through time (Fig. 3b) for lineages A and B. We observed a fast epidemic growth of lineage A following its introduction into France in approximately 1999. Lineage A reached a maximum median *R*_e_ of two, up until the introduction of lineage B around 2005. From 2005 to 2009, lineage A epidemic declined rapidly concurrently with the fast epidemic growth of lineage B that reached a maximum median *R*_e_ of 2.5. This period was then followed by a sharp decline in the lineage B epidemic between 2009 and 2012, followed by an epidemic resurgence in 2013. This trend may be due to competition between the French *M. bovis* st2 lineage B strains and the first *M. bovis* st3 isolates detected in France at the same period. The absence of coalescence events in lineage B after 2015 in the phylogeny does not provide grounds for confidently estimating epidemic dynamics after 2015. In addition, the median become-uninfectious rate parameters estimated for lineages A and B were 0.2 and 0.26, respectively. These values are expressed as units per year and reflect the inverse of the time of infectiousness, which was 1825 days (5 years) for lineage A and 1404 days (3.85 years) for lineage B. This shorter time of infectiousness of lineage B could reflect better transmissibility and thus explain how lineage B progressively supplanted lineage A between 2005 and 2010.

### Polymorphisms associated with lineages

**Fig. 4.**
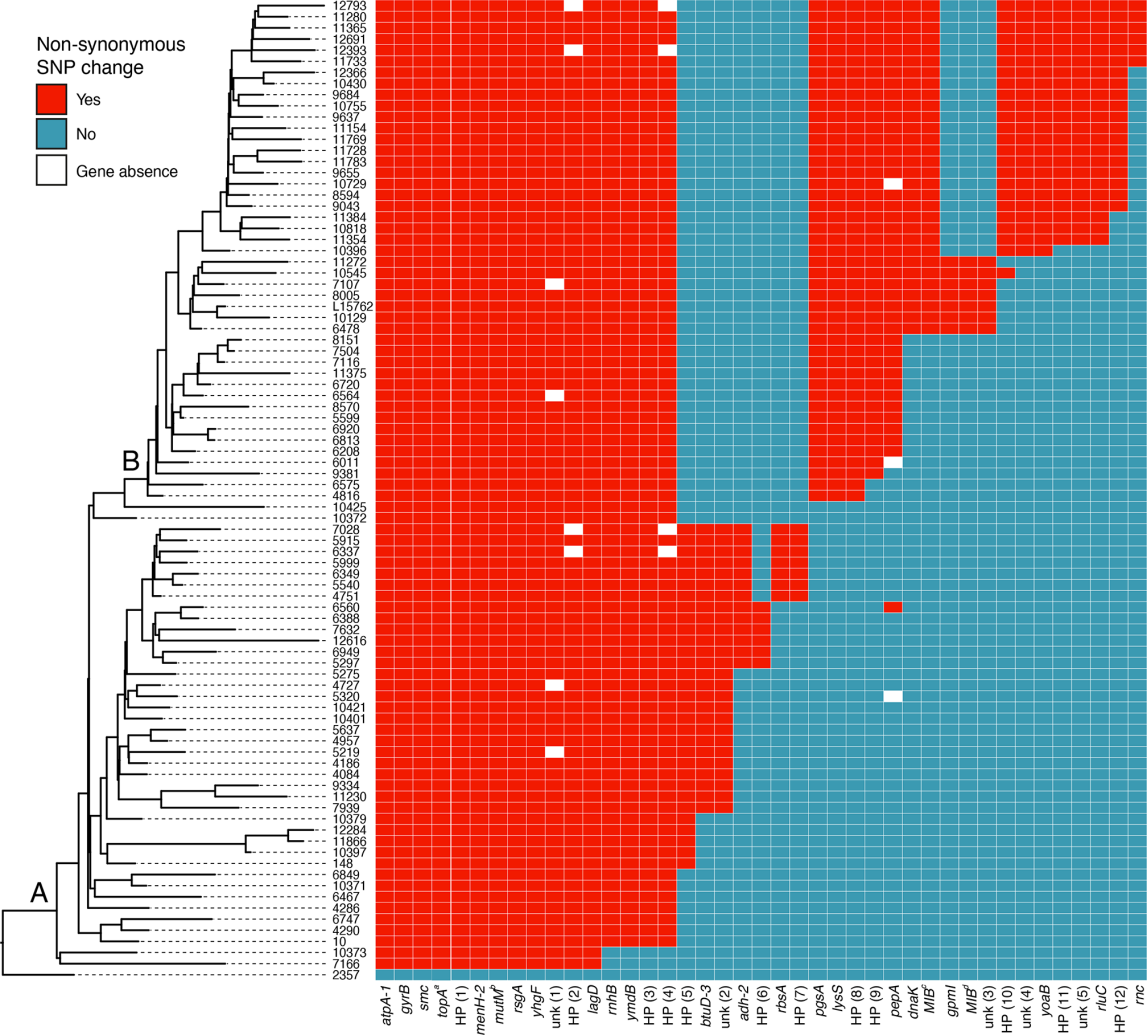
Main non-synonymous SNP changes identified among the French *Mycoplasma bovis* subtype 2 isolates. Left panel shows the French *M. bovis* subtype 2 phylogeny with lineages A and B ancestral node-annotated. Right panel shows non-synonymous SNP changes comparatively to the ancestral isolate 2357, with corresponding gene names given at the bottom of the panel. Superscript letters associated with gene names correspond to several mutations at different site positions: ‘a’ denotes mutations in the four site positions identified in *topA*, ‘b’ denotes the three site positions identified in *mutM* and ‘c’ and ‘d‘ denote site positions 1550 and 1852 identified in *MIB*, respectively. White blocks indicate gene absences (or possibly non-recovery in sequencing) in certain lineages due to gene deletion in genome isolates. Non-synonymous SNPs that could have an impact on gene functions are detailed in Table S2.

Based on the pan-genome, we searched for non-synonymous mutations that can be associated with lineages A or B (and their sub-lineages) observed along the French *M. bovis* st2 phylogeny (Fig. 2). Out of the 891 genes of the pan-genome, 424 had one or several non-synonymous mutations. Among these genes, we chose to focus solely on mutations shared by phylogenetic monophyletic clusters of at least six isolates, i.e. 46 non-synonymous sites in 40 genes (Table S2). Fig. 4 plots these non-synonymous sites and associated genes along the French *M. bovis* st2 phylogenetic tree. In comparison to strain 2357 collected in 2000 that did not belong to a large epidemic in France, isolates belonging to lineages A and B can be described and distinguished by a pool of mutated genes. Some of these mutations are located in functional domains of proteins. For instance, *topA* and *mutM*, which encode a type-I DNA topoisomerase and a DNA-formamidopyrimidine glycosylase, respectively, possess the greatest number of substitutions in domains related to their functions (Fig. 4 and Table S2). Within lineage A, we observed seven aa changes in five different monophyletic groups. Twelve isolates had an aa change at position 316 in *adh-2* (alcohol dehydrogenase), an aa positioned next to tetramere interface residue 315 of the protein involved in oxydoreductase activity. Lineage B was characterized by aa changes in 10 genes, including *MIB* (* Mycoplasma* Ig-binding protein) and *dnaK* (protein chaperone belonging to the heat-shock protein 70 family), which play key roles in immune evasion and virulence [51–54] (Fig. 4 and Table S2). The upper segment of lineage B that comprises the most recent isolates is defined by aa change in eight genes where substitutions affect three sugar or glycerol ABC transporters, one rRNA processing isomerase and one ribonuclease (Fig. 4 and Table S2).

## Discussion

### Sample collection and generated genomic data are suitable for phylodynamic inferences

Phylodynamic approaches have so far rarely been applied to bacterial pathogens due to inherent methodological limitations [5], such as large and slow-evolving genomes and high levels of recombination and homoplasy. Here we used *M. bovis* as a model to investigate how these approaches based on genomic data could be used to improve the monitoring of pathogens in the context of passive veterinary surveillance. To do so, we selected 88 French *M. bovis* st2 isolates collected over almost 20 years via the Vigimyc network, and sequenced their genomes using an optimized Illumina workflow. We deliberately focused on *M. bovis* st2, as it is the majority subtype currently circulating in France and for which we had sufficient data. The exclusion of other *M. bovis* subtypes present in France during our sampling period is a clear limitation of our study, as it does not give a complete epidemiological picture. Furthermore, we cannot exclude that due to our sampling scheme, which is based on passive surveillance data, some minor genetic lineages may have been missed outside the two main French cattle farming regions we sampled. Nevertheless, the strength of the study relies on our regular temporal sampling required for robust phylodynamic inferences [25].

Despite fragmented draft genome assemblies, pan-genome analysis of the French *M. bovis* st2 isolates revealed a core genome of 656 genes (i.e. approximately 70 % of the total *M. bovis* genome size). After curation, we selected 531 genes to serve as the basis for phylodynamic analyses, which is slightly more than the 478 genes selected for a recent core genome MLST analysis of the whole *M. bovis* species [55]. The *M. bovis* st2 core-genome alignment contained few homoplasic sites, with a consistency index on parsimony-informative sites with the associated phylogeny of 0.88. This suggests a low impact of homologous recombination on time-course evolution of the *M. bovis* st2 genome. In addition, the robust and high estimated mean clock rate of 4.6×10^−6^ substitutions per site per year for this *M. bovis* population fits with the range previously obtained in the genus *Mycoplasma *, i.e. ~1×10^−5^ substitutions per site per year for *M. gallisepticum* over a 13 year period [9] and 5×10^−7^ substitutions per site per year for *Mycoplasma mycoides* subsp. *mycoides* over several centuries [56]. The characteristics of genomic evolution in *Mycoplasma* species and especially the *M. bovis* st2 population studied here enabled well-supported phylogenetic and molecular clock analyses and relevant coalescent and birth–death-based demographic and epidemic inferences, which account for potential sampling biases [40, 48]. Nevertheless, despite our substantial genomic sampling, we did not find a spatial structure in the French *M. bovis* st2 population, even though cattle populations and sectors are spatially structured within the country. A similar observation was made by Bokma *et al.* [19] in Belgium at both the spatial and sector scales (i.e. among diary, beef and veal farms). Cattle trade networks within national boundaries are dense and often the source of long-distance dispersal. Studying diffusion patterns at country level would definitively need an even larger sample size.

### Multiple introductions of *M. bovis* st2 lineages into France and their epidemic dynamics

Genome-wide phylodynamic analyses gave a refined timeline of critical events in the evolutionary and epidemic dynamics of *M. bovis* st2, the current dominant circulating subtype in France. Our results indicate that two lineages, likely originating from successful independent introductions of international *M. bovis* st2 strains, have successively been dominant in France (Fig. 2 and S2). There was also a preliminary introduction in the surveillance data, with isolate no. 2357 collected in 2000, but it did not result in a large-scale, long-lasting epidemic. The first dominant lineage, which we called lineage A, replaced a pre-existing *M. bovis* st1 population [16] from around 2000. It has been hypothesized that the increase in the frequency of st2 was driven by selection and substantially facilitated by its higher resistance to most antibiotics compared to st1 strains [16, 22]. This emergence resulted in a diversification of lineage A (Fig. 2), and demographic analyses (Fig. 3) suggest that it reached its maximum *N*_e_ in around 2005. Lineage A then gradually declined from 2005 and became a minority *M. bovis* st2 variant in France after 2010. This was observable through the Vigimyc surveillance system, where lineage A strains drastically decreased in frequency in our sampling after 2010. Lineage A was progressively replaced by a new *M. bovis* st2 lineage that arose around 2005, which we called lineage B. This second *M. bovis* st2 lineage took around 10 years to replace lineage A and reach its maximum *N*_e_ around 2015.

### Different transmission rates of *M. bovis* lineages

Such lineage replacement in a large population is unlikely to be a neutral selection process. Indeed, our fitness analysis shows that the basal branches of lineage B have better fitness than the contemporaneous branches in lineage A. In a previous study, it was shown that *M. bovis* st2 isolates are very homogeneous in their resistance phenotypes (loss of susceptibility to most antimicrobial families except fluoroquinolones) and genotypes [57]. This, along with an inferred shorter duration of infection of lineage B, suggests that lineage B may have a higher transmission rate than lineage A. Recent models have investigated how these differences in transmission or duration of infection can lead to replacement of pathogen populations [58]. This may also explain why the pace at which *N*_e_ and *R*_e_ increased for lineages A and B was relatively similar, whereas the first replacement (of *M. bovis* st1) involving lineage A was likely driven by selection for antibiotic resistance [16]. Further investigations are necessary, but we managed to identify several amino acid changes that occurred in the French *M. bovis* st2 core-genome at the root of lineage B that may provide candidate genes to explain a potential increase in transmissibility. In particular, some of these non-synonymous mutations appeared in genes that play key roles in immune evasion and virulence, such as *MIB* and *dnaK*. We need to understand the architecture and level of transmission required to drive a replacement of circulating lineages in order to deal with future epidemiological situations. Moreover, within lineage B, we identified an instability of the population, illustrated by the decline in *R*_e_ between 2009 and 2012 before a second phase of increase. Based on the Vigimyc French surveillance data, *M. bovis* st3 isolates were first identified in France in 2011 and currently account for up to 20 % of the circulating *M. bovis* population [18, 23]. The dynamics of the lineage B population might have been impacted by the emergence of this *M. bovis* st3 lineage in the French cattle population. In this context, two hypotheses might explain the observed rebound of lineage B after 2012: an independent introduction of a new *M. bovis* st2 lineage that was fitter than circulating lineage B, or an adaptation arising in lineage B to the new epidemiological environment involving *M. bovis* st3. It would be instructive to extend the epidemic dynamic analyses to all the subtypes circulating in France in order to understand the global dynamics and potential interactions between lineages, especially if they also show patterns of substitution and recombination compatible with genome-wide phylodynamic analyses.

### Defining a strategy to characterize *M. bovis* lineages and dynamics in France and globally

Our study argues strongly for using the *polC* gene fragment as a reliable subtyping marker, as it has few discrepancies with the genomic data (Fig. S2), possibly due to homologous recombination or mycoplasma chromosomal transfer events [33, 59]. However, our results also show that within one subtype, the *polC* marker failed to identify lineages, and their dynamics of evolution and replacement. New markers are needed for deeper epidemiological monitoring of *M. bovis* subtypes in France. Identified genes with mutations (Fig. 4 and Table S2) specific to lineage A or B (or their sub-lineages) would make good candidates for designing discriminant markers to improve the sensitivity of *M. bovis* st2 surveillance in the Vigimyc network. At the international level, it is common to find different subtypes of *M. bovis* circulating within the same country [18]. Although MLST data, such as the Register *et al.* scheme [15], have better discriminatory ability than the *polC* locus, they still lack sufficient phylogenetic resolution to identify and infer population dynamics of lineages within subtypes. A genomic investigation of the dynamics of the main subtypes worldwide would help to clarify the global dynamics of *M. bovis* and the potential interactions between the incidence and prevalence of circulating subtypes. After assigning the main circulating subtypes by typing a few loci, it would then be necessary to sequence just a few isolates per lineage per year in order to update information on their respective dynamics and identify potential interactions. Genomic data would also help to clarify the impact of imported genotypes on *M. bovis* epidemiology between and within countries. The study published by Yair *et al.* [21], which highlighted the diversity of *M. bovis* genotypes carried by imported animals, illustrates the numerous potential introduction routes to occur. Among others, the emergence of *M. bovis* in New Zealand [60] and the emergence of *M. bovis* st2 and st3 lineages in France offer clear examples that such introductions can give imported genotypes a foothold from which to circulate rapidly. The accumulation of genomes from Eastern European countries and Israel near the roots of French *M. bovis* st2 lineages A and B illustrate shared ancestry and traces of international migrations (Fig. S2). Similarly, the interleaved branching of French and Spanish strains in the most recent part of lineage B might also signal recurrent exchanges (Fig. S2). Within lineages, a better genomic description of *M. bovis* populations circulating in different countries that trade animals would be useful for surveillance and management.

### Conclusion and perspectives

In this study, we showed that the emergence of fitter and potentially more transmissible genotypes from international importations (and possibly local adaptations) may be an important driver of *M. bovis* population evolution. The study also highlights that genome-wide phylodynamic approaches have the potential to transform our understanding of the spatiotemporal and population dynamics of *M. bovis* and, by extension, any *Mycoplasma* species with reasonably low recombination rates. Such phylodynamic approaches could play a central role in the design of more effective surveillance systems and disease control measures, as well as for assessing the impact of future vaccination strategies. Nevertheless, the power of phylodynamic analyses is still hampered by the lack of genomic data. A genome-based surveillance must therefore accompany the development of phylodynamic methods to monitor *M. bovis* dynamics more accurately and precisely and thus better support the estimation of future trends and disease burden.

## Supplementary Data

Supplementary material 1
