# Supplementary material for: Genome-wide phylodynamic approach reveals the epidemic dynamics of the main Mycoplasma bovis subtype circulating in France
Source: Microb Genom. 2023 Jul 24;9(7):mgen001067. doi: 10.1099/mgen.0.001067 (PMC10438803; doi:10.1099/mgen.0.001067)
Supplement: Supplementary material 1 [file mgen-9-1067-s001.pdf]

Table S1. *Mycoplasma bovis* samples: information and sequencing statistics

BioSample accession	SRA accession	Isolate name	Sampling location (Region)	Collection date	Sampling type	Nb reads after QC	% reads mapped (ref: NC_014760)	% genomic coverage (ref: NC_014760)	Mean depth coverage (ref: NC_014760)	Total bases denovo draft assembly	nb scaffolds denovo draft assembly	N50 denovo draft assembly	Nb CDS annotated denovo draft assembly
SAMN29794518	SRR20305711	10	France: Manche (North-West)	28-Apr-2005	Bronchoalveolar lavage	348454	98,97	94.9313	44.6293	950492	574	14095	741
SAMN29794519	SRR20305710	148	France: Mayenne (North-West)	28-Jan-2005	Lung parenchyma	266562	96,20	96.1146	35.2244	980761	424	15278	786
SAMN29794520	SRR20305671	2357	France: Aveyron (Center)	10-Apr-2000	Lung parenchyma	228212	96,17	95.4449	29.7101	977748	468	12701	779
SAMN29794521	SRR20305660	4084	France: Cantal (Center)	26-Dec-2005	Lung parenchyma	327752	96,21	95.0226	41.6097	971267	419	16562	776
SAMN29794522	SRR20305705	4186	France: Allier (Center)	6-Jan-2006	Bronchoalveolar lavage	248672	95,91	95.1398	31.3787	932658	420	8223	765
SAMN29794523	SRR20305694	4286	France: Mayenne (North-West)	20-Feb-2006	Lung parenchyma	353842	96,69	95.0069	44.5201	940229	244	10612	774
SAMN29794524	SRR20305683	4290	France: Seine-Maritime (North-West)	18-Feb-2006	Lung parenchyma	302254	95,85	95.9269	37.2749	954765	269	11463	775
SAMN29794525	SRR20305644	4727	France: Saone-et-Loire (Center)	8-Jan-2007	Bronchoalveolar lavage	260468	95,65	94.004	32.8542	949523	452	8847	772
SAMN29794526	SRR20305633	4751	France: Aveyron (Center)	30-Jan-2007	Lung parenchyma	245696	96,12	91.9329	30.9321	930574	437	11259	749
SAMN29794527	SRR20305624	4816	France: Seine-Maritime (North-West)	26-Mar-2007	Lung parenchyma	237330	96,16	94.9316	32.4126	953048	282	8301	781
SAMN29794528	SRR20305709	4957	France: Morbihan (North-West)	5-Aug-2007	Lung parenchyma	292024	96,31	95.402	36.6922	968325	376	16603	781
SAMN29794529	SRR20305680	5219	France: Mayenne (North-West)	21-Dec-2008	Lung parenchyma	252494	96,20	94.398	34.4694	950356	259	19942	777
SAMN29794530	SRR20305679	5275	France: Maine-et-Loire (North-West)	25-Mar-2008	Lung parenchyma	305662	95,69	93.6252	40.1848	948392	341	13020	768
SAMN29794531	SRR20305678	5297	France: Saone-et-Loire (Center)	14-Apr-2008	Lung parenchyma	234334	96,37	92.0334	30.228	935433	398	14746	750
SAMN29794532	SRR20305677	5320	France: Seine-Maritime (North-West)	9-May-2008	Bronchoalveolar lavage	256200	96,05	93.3748	32.9459	947849	448	11630	758
SAMN29794533	SRR20305676	5540	France: Allier (Center)	8-Oct-2008	Bronchoalveolar lavage	269354	96,40	94.4566	33.9977	970877	531	13036	773
SAMN29794534	SRR20305675	5599	France: Manche (North-West)	10-Nov-2008	Lung parenchyma	317104	96,35	95.939	40.1703	983029	492	11735	785
SAMN29794535	SRR20305674	5637	France: Cote-dOr (Center)	30-Dec-2008	Lung parenchyma	245622	96,26	94.8756	33.4601	961481	346	13847	775
SAMN29794536	SRR20305673	5915	France: Allier (Center)	2-Feb-2009	Bronchoalveolar lavage	210430	99,33	92.0569	29.7848	891562	247	16187	726
SAMN29794537	SRR20305672	5999	France: Orne (North-West)	10-Feb-2009	Lung parenchyma	268366	99,40	94.3141	36.9955	920361	292	15714	749
SAMN29794538	SRR20305670	6011	France: Saone-et-Loire (Center)	27-Mar-2009	Nasopharyngeal swab	224918	99,19	91.5677	30.5106	868722	235	10470	707
SAMN29794539	SRR20305669	6208	France: Mayenne (North-West)	9-Oct-2009	Bronchoalveolar lavage	308348	96,65	96.1889	42.3095	967574	251	18828	792
SAMN29794540	SRR20305668	6337	France: Aveyron (Center)	17-Dec-2009	Lung parenchyma	262428	95,83	93.6769	35.2865	947416	333	8771	774
SAMN29794541	SRR20305667	6349	France: Seine-Maritime (North-West)	29-Dec-2009	Bronchoalveolar lavage	311788	96,30	94.0191	42.5224	947945	266	16248	774
SAMN29794542	SRR20305666	6388	France: Orne (North-West)	7-Jan-2010	Lung parenchyma	300526	99,39	94.7632	40.6641	922479	275	14212	749
SAMN29794543	SRR20305665	6467	France: Allier (Center)	21-Mar-2010	Lung parenchyma	289302	95,79	94.8926	38.3231	952495	239	15271	779
SAMN29794544	SRR20305664	6478	France: Aveyron (Center)	1-Apr-2010	Lung parenchyma	295668	96,17	95.6435	39.2436	962759	271	16604	777
SAMN29794545	SRR20305663	6560	France: Dordogne (Center)	19-May-2010	Lung parenchyma	294630	95,99	95.4082	36.2467	950764	306	11504	772
SAMN29794546	SRR20305662	6564	France: Vendee (North-West)	20-May-2010	Lung parenchyma	282086	95,98	94.7111	35.6367	938492	258	8833	783
SAMN29794547	SRR20305661	6575	France: Correze (Center)	14-May-2010	Lung parenchyma	289952	95,77	94.5442	38.2239	943845	244	16072	765
SAMN29794548	SRR20305659	6720	France: Mayenne (North-West)	17-Dec-2010	Nasopharyngeal swab	316100	95,75	96.0354	41.8649	964296	286	12485	791
SAMN29794549	SRR20305658	6747	France: Sarthe (North-West)	28-Jan-2011	Nasopharyngeal swab	338424	79,36	94.2613	36.7878	932373	267	8012	760
SAMN29794550	SRR20305657	6813	France: Manche (North-West)	13-Apr-2011	Bronchoalveolar lavage	249928	95,96	95.787	31.9701	951260	273	11034	777
SAMN29794551	SRR20305656	6849	France: Vendee (North-West)	28-Apr-2011	Lung parenchyma	350654	98,97	94.9735	45.8367	914310	275	12395	744
SAMN29794552	SRR20305655	6920	France: Seine-Maritime (North-West)	10-May-2011	Lung parenchyma	359972	95,65	96.1105	47.6816	974985	330	12352	783
SAMN29794553	SRR20305654	6949	France: Indre (Center)	27-May-2011	Bronchoalveolar lavage	288756	96,20	94.1918	37.6826	937668	277	14792	765
SAMN29794554	SRR20305653	7028	France: Loire (Center)	23-Sep-2011	Lung parenchyma	267832	96,23	90.6286	35.2853	914420	305	9545	740
SAMN29794555	SRR20305708	7107	France: Loire (Center)	3-Jan-2012	Lung parenchyma	263680	96,24	94.6158	32.3951	951094	308	16482	769
SAMN29794556	SRR20305707	7116	France: Calvados (North-West)	20-Jan-2012	Lung parenchyma	264110	95,98	95.8164	33.1052	977284	475	10343	783
SAMN29794557	SRR20305706	7166	France: Aveyron (Center)	13-Feb-2012	Lung parenchyma	249774	96,54	94.47	31.3878	958260	382	12493	787
SAMN29794558	SRR20305704	7504	France: Mayenne (North-West)	25-Oct-2012	Lung parenchyma	379082	96,19	95.945	47.0642	967703	338	11894	788
SAMN29794559	SRR20305703	7632	France: Loire-Atlantique (North-West)	26-Nov-2012	Lung parenchyma	258084	99,06	91.8879	35.0052	885024	246	9508	725
SAMN29794560	SRR20305702	7939	France: Manche (North-West)	6-Mar-2013	Nasopharyngeal swab	401790	96,19	94.9004	49.3991	946370	313	10231	773
SAMN29794561	SRR20305701	8005	France: Seine-Maritime (North-West)	23-Mar-2013	Lung parenchyma	313544	96,00	95.435	39.3757	972296	454	11106	781

SAMN29794562	SRR20305700	8149	France: Cote-dOr (Center)	18-May-2013	Lung parenchyma	309878	92,03	93.3532	37.9133	946592	400	4964	752
SAMN29794563	SRR20305699	8151	France: Dordogne (Center)	13-May-2013	Lung parenchyma	355038	96,11	96.0921	45.4441	983200	440	14087	783
SAMN29794564	SRR20305698	8570	France: Finistere (North-West)	9-Dec-2013	Lung parenchyma	280512	96,43	95.7191	36.5177	951622	276	10636	779
SAMN29794565	SRR20305697	8594	France: Manche (North-West)	23-Dec-2013	Lung parenchyma	342648	95,57	95.4357	42.0397	958324	338	10870	783
SAMN29794566	SRR20305696	9043	France: Cantal (Center)	17-Mar-2014	Nasopharyngeal swab	311792	95,47	95.045	38.1269	992903	606	8095	778
SAMN29794567	SRR20305695	9334	France: Loire (Center)	9-Sep-2014	Lung	308128	95,79	93.3728	37.7411	921508	268	9604	761
SAMN29794568	SRR20305693	9381	France: Cotes-dArmor (North-West)	10-Oct-2014	Bronchoalveolar lavage	309080	98,99	95.0928	39.3628	911040	233	11851	745
SAMN29794569	SRR20305692	9637	France: Finistere (North-West)	7-Jan-2015	Lung parenchyma	232154	96,23	94.8579	30.5604	941665	260	10310	775
SAMN29794570	SRR20305691	9655	France: Saone-et-Loire (Center)	31-Jan-2015	Lung parenchyma	357264	96,35	95.6135	43.1088	980631	512	13560	778
SAMN29794571	SRR20305690	9684	France: Morbihan (North-West)	4-Feb-2015	Lung parenchyma	283902	96,34	95.381	36.4051	949979	251	11610	774
SAMN29794572	SRR20305689	10129	France: Allier (Center)	27-Jul-2015	Lung parenchyma	294786	96,16	94.4835	38.3135	949992	318	8954	771
SAMN29794573	SRR20305688	10371	France: Haute-Vienne (Center)	11-Dec-2015	Lung	290452	96,34	96.2068	38.3801	975713	383	11678	780
SAMN29794574	SRR20305687	10372	France: Haute-Vienne (Center)	23-Feb-2005	Lung	431050	96,03	95.5377	50.6743	1027640	953	16247	779
SAMN29794575	SRR20305686	10373	France: Creuse (Center)	9-Mar-2005	Lung	348798	97,50	95.1805	43.0862	958374	385	20274	766
SAMN29794576	SRR20305685	10379	France: Dordogne (Center)	3-Aug-2005	Lung	313774	96,24	95.3932	40.8189	971548	393	15282	778
SAMN29794577	SRR20305684	10396	France: Haute-Vienne (Center)	19-Jun-2012	Lung	307940	99,49	95.1687	41.619	940709	465	11962	742
SAMN29794578	SRR20305682	10397	France: Haute-Vienne (Center)	12-Feb-2014	Lung	295304	95,67	95.1889	38.706	961844	331	10816	783
SAMN29794579	SRR20305681	10401	France: Haute-Vienne (Center)	5-Mar-2007	Nasopharyngeal swab	285404	95,40	92.1342	35.4774	933568	433	9546	756
SAMN29794580	SRR20305652	10421	France: Haute-Vienne (Center)	8-Oct-2007	Bronchoalveolar lavage	363740	96,07	92.7868	45.1711	956465	525	18277	753
SAMN29794581	SRR20305651	10425	France: Indre (Center)	25-Feb-2015	Lung parenchyma	358280	95,94	95.311	48.0116	970803	397	11812	776
SAMN29794582	SRR20305650	10430	France: Maine-et-Loire (North-West)	7-Dec-2015	Bronchoalveolar lavage	291250	96,07	96.8358	39.0187	1025470	650	17254	792
SAMN29794583	SRR20305649	10545	France: Saone-et-Loire (Center)	28-Jan-2016	Bronchoalveolar lavage	287136	96,24	95.7361	38.2182	980066	398	13837	781
SAMN29794584	SRR20305648	10729	France: Ille-et-Vilaine (North-West)	23-Mar-2016	Lung parenchyma	279340	95,88	93.8522	34.9507	949417	460	8271	771
SAMN29794585	SRR20305647	10755	France: Loire (Center)	4-Apr-2016	Lung parenchyma	299210	96,29	95.0711	37.2959	941848	259	9229	762
SAMN29794586	SRR20305646	10818	France: Cantal (Center)	26-Apr-2016	Lung parenchyma	261042	99,08	93.3742	33.7642	886699	270	9032	736
SAMN29794587	SRR20305645	11154	France: Morbihan (North-West)	4-Nov-2016	Lung parenchyma	249520	96,33	95.1522	33.444	945677	257	8336	777
SAMN29794588	SRR20305643	11230	France: Orne (North-West)	9-Dec-2016	Bronchoalveolar lavage	286154	96,14	93.7494	36.7719	956523	465	8664	769
SAMN29794589	SRR20305642	11272	France: Aveyron (Center)	4-Jan-2017	Lung parenchyma	282494	99,45	93.5233	35.2598	921818	563	7429	739
SAMN29794590	SRR20305641	11280	France: Mayenne (North-West)	6-Jan-2017	Lung parenchyma	360626	96,22	95.5289	43.1288	962024	338	14964	787
SAMN29794591	SRR20305640	11354	France: Mayenne (North-West)	24-Feb-2017	Lung parenchyma	323856	96,23	94.7851	40.4295	941812	257	14120	779
SAMN29794592	SRR20305639	11365	France: Loire (Center)	28-Feb-2017	Lung parenchyma	387690	96,19	95.4041	47.6435	955038	317	8132	778
SAMN29794593	SRR20305638	11375	France: Manche (North-West)	6-Mar-2017	Lung parenchyma	235170	96,88	95.2352	31.6861	962328	359	13636	783
SAMN29794594	SRR20305637	11384	France: Vendee (North-West)	13-Mar-2017	Lung parenchyma	258948	96,42	95.1069	33.9158	947931	319	8092	779
SAMN29794595	SRR20305636	11728	France: Loire-Atlantique (North-West)	2-Jan-2018	Lung parenchyma	267950	95,91	95.2928	35.5671	947571	258	9875	777
SAMN29794596	SRR20305635	11733	France: Orne (North-West)	11-Jan-2018	Nasopharyngeal swab	328952	96,05	95.3346	39.4728	960448	372	9131	789
SAMN29794597	SRR20305634	11769	France: Seine-Maritime (North-West)	25-Jan-2018	Lung parenchyma	254408	99,10	94.4954	32.9949	906212	238	14179	733
SAMN29794598	SRR20305632	11783	France: Creuse (Center)	7-Feb-2018	Lung parenchyma	295994	96,08	95.5742	38.6446	967909	374	16247	777
SAMN29794599	SRR20305631	11866	France: Haute-Vienne (Center)	28-Mar-2018	Lung parenchyma	259898	95,64	94.9586	34.3036	947556	278	8123	786
SAMN29794600	SRR20305630	12284	France: Haute-Vienne (Center)	8-Jan-2019	Lung parenchyma	290738	95,62	94.7354	36.439	919623	179	10976	774
SAMN29794601	SRR20305629	12366	France: Cotes-dArmor (North-West)	3-Feb-2019	Lung parenchyma	261832	96,26	95.0385	32.9577	938102	277	9762	774
SAMN29794602	SRR20305628	12393	France: Aveyron (Center)	20-Feb-2019	Lung parenchyma	252944	99,43	94.1785	34.5073	903953	221	9930	736
SAMN29794603	SRR20305627	12616	France: Seine-Maritime (North-West)	5-Jun-2019	Lung parenchyma	246122	96,80	93.2886	31.8357	933425	311	8072	764
SAMN29794604	SRR20305626	12691	France: Puy-de-Dome (Center)	21-Sep-2018	Lung parenchyma	248248	96,84	94.5689	33.1048	955955	303	16524	770
SAMN29794605	SRR20305625	12793	France: Ille-et-Vilaine (North-West)	9-Nov-2019	Lung parenchyma	346648	96,16	94.3753	44.1302	945202	287	9962	779

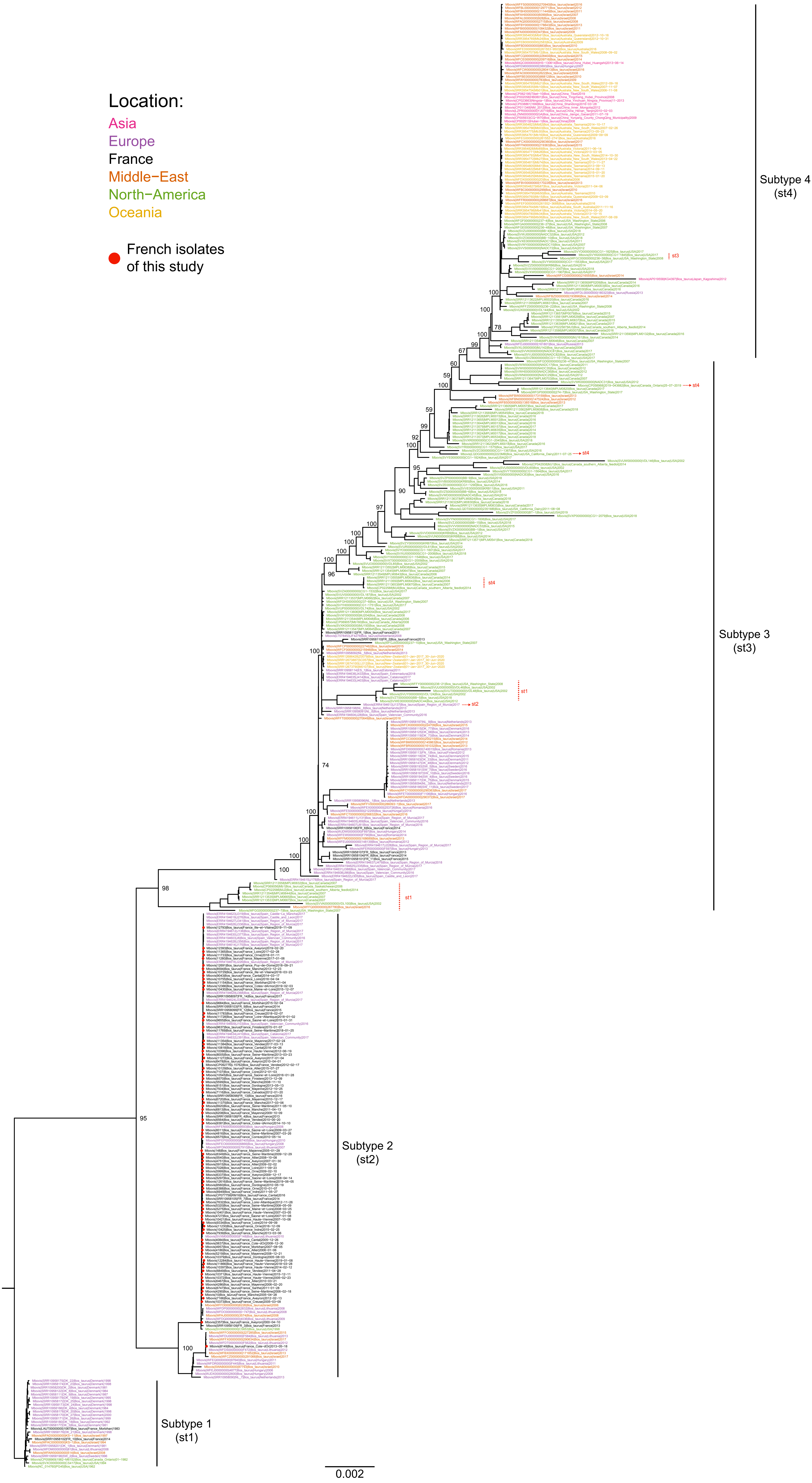

Fig. S1. Global genetic diversity of *Mycoplasma bovis*. Maximum likelihood phylogenetic tree based on a core-genome alignment combining the 88 French *M. bovis* subtype 2 genomes generated in this study and 339 publicly-available subsampled *M. bovis* genomes. The phylogeny is rooted on the oldest subtype (subtype 1). Statistical support for nodes were assessed using an ultrafast bootstrap (1000 replicates). Tips are colored according to the sample continent or country of origin. Red colored circles at tips denote French *M. bovis* subtype 2 genomes generated in this study. Solid bars at right correspond to consistent subtyping results between the core-genome phylogeny and *polC* gene fragment sequence. Red dotted bars and arrows correspond to inconsistencies in subtyping results between the core-genome phylogeny and *polC* fragment sequence.

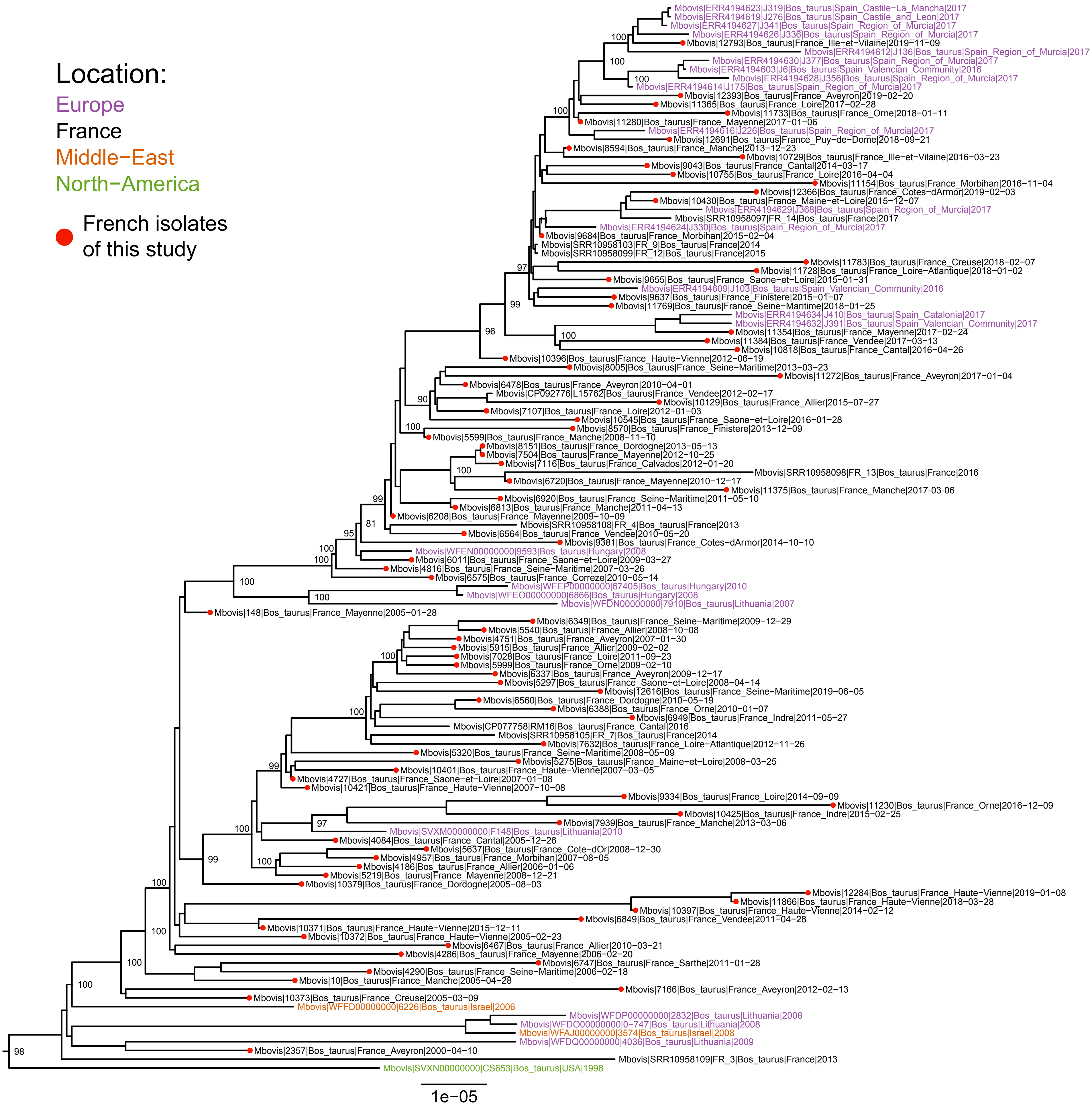

Fig. S2. Genetic diversity of the dominant *Mycoplasma bovis* subtype 2 lineage. Maximum likelihood phylogenetic tree shown in Figure S1, zoomed in on the dominant *M. bovis* subtype 2 lineage.

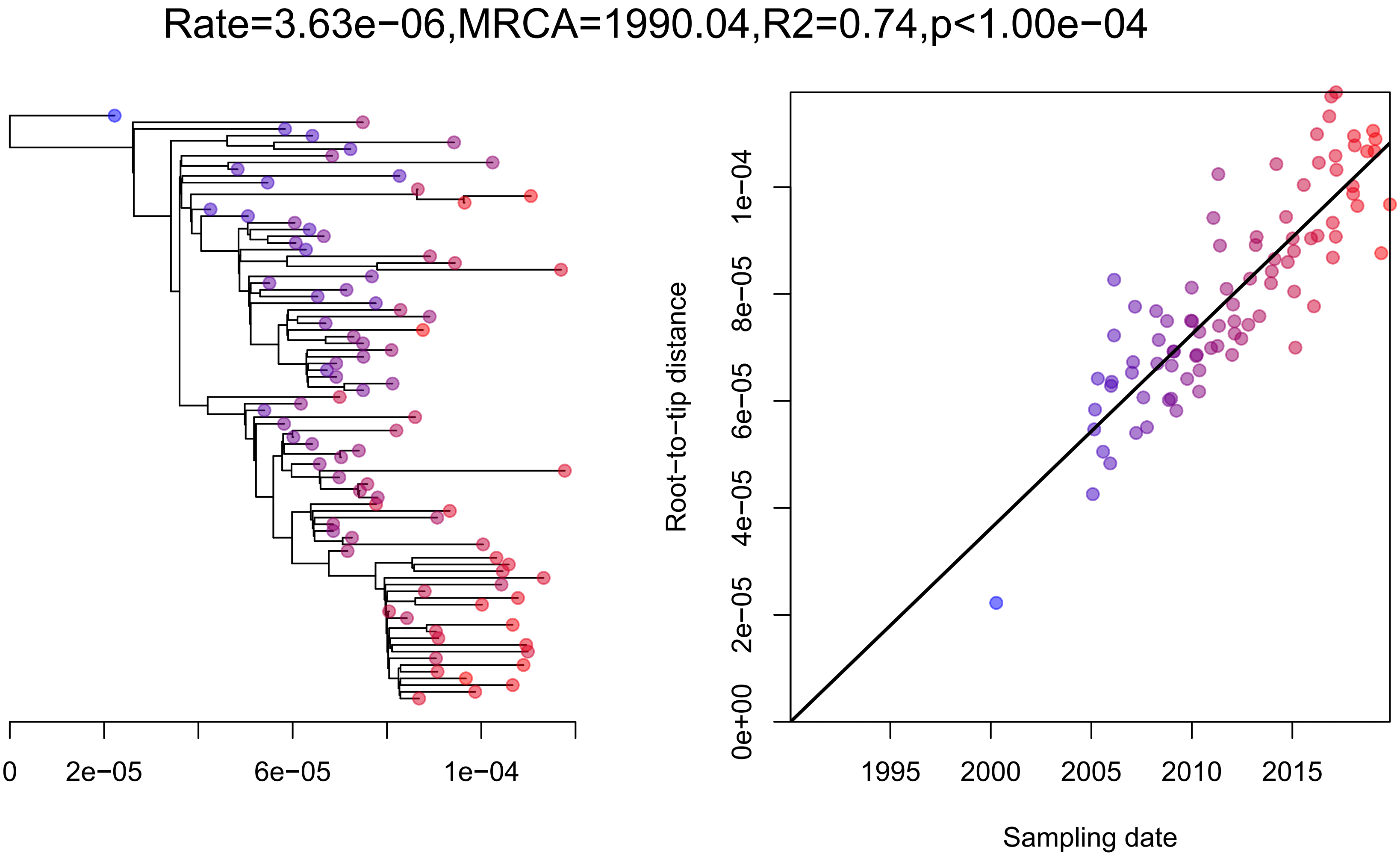

Fig. S3. Bactdating root-to-tip results. Left panel shows the maximum likelihood French *Mycoplasma bovis* subtype 2 phylogeny based on a core-genome alignment of 88 genomes. Tip circles are colored according to isolate sampling dates. Right panel shows the regression analysis between the sampling dates and the root-to-tip distances in the phylogenetic tree.

Table S2. Non-synonymous SNP changes identified among the French *Mycoplasma bovis* subtype 2 isolates, and their potential impact on gene function (see Figure 4). Nucleotide and amino-acid reference refer to *M. bovis* 2357 isolate sequence. Prediction of functional domain was performed using interproscan. Different amino-acid classes are differentiated by colors

Gene	Gene Name	Nt position	Nt reference	Nt alternative	aa position / aa total	aa reference	aa alternative	Impact on protein domain
atp-1	ATP synthase subunit alpha	1360	T	A	454/528	W	R	ATP synthase alpha/beta chain, C terminal domain (PF00306)
gyrB	DNA gyrase subunit B	1594	A	T	532/655	T	S	toprim domain, conserved in DNA primase (PF01751)
smc	structural maintenance of chromosome protein	1108	A	C	370/992	I	L	smc hinge domain
topA	type I DNA topoisomerase	1702	T	A	568/617	S	T	topoisomerase DNA binding C4 zinc finger (PF01396)
		1730	C	T	577/617	A	V	
		1734	G	A	578/617	M	I	
		1746	C	A	582/617	N	K	
HP (1)	hypothetical protein	38	T	C	10/232	F	S	signal peptide
menH-2	2-succinyl-6-hydroxy-2,4-cyclohexadiene-1-carboxylate synthase	602	C	T	201/266	T	I	hydrolase 4 domain (PF012146)
mutM	DNA-formamidopyrimidine glycosylase	209	G	A	70/279	S	N	formamidopyrimidine-DNA glycosylase N-terminal domain (PF01149)
		212	C	T	71/279	S	L	
		215	C	T	72/279	T	I	
rsgA	small ribosomal subunit biogenesis GTPase	238	T	C	80/281	Y	H	circularly permuted (CP)-type guanine nucleotide-binding (G) domain (IPR030378)
yhgF	RNA (S1 domain)-binding protein	1471	G	C	491/719	D	H	helix-hairpin-helix motif (PF12836)
unk (1)	unknown DUF31 family protein	427	C	T	143/579	H	Y	non cytoplasmic domain
HP (2)	hypothetical protein	23	T	C	8/52	I	T	no domain impacted
lagD	ABC transporter ATP-binding protein	1835	G	A	612/685	G	D	circularly permuted (CP)-type guanine nucleotide-binding (G) domain (IPR030378)
rnhB	ribonuclease HII	260	A	T	87/207	K	M	RNA/DNA hybrid binding site (aa 85 to aa 87)
ymdB	2',3'-cyclic-nucleotide 2'-phosphodiesterase	742	G	A	248/270	S	G	no domain impacted
HP (3)	hypothetical protein	643	C	T	215/856	L	F	no domain impacted
HP (4)	hypothetical protein	271	G	A	91/151	A	T	no domain impacted
HP (5)	hypothetical protein	131	C	T	44/420	T	I	transmembrane domain
btuD-3	vitamin B12 import ATP-binding protein	112	A	A	37/539	E	D	no domain impacted
unk (2)	unknown ABC transporter permease	1829	C	T	610/2707	P	L	non cytoplasmic domain
adh-2	alcool deshydrogenase	947	G	A	316/353	G	E	tetramere interface in residu 315
HP (6)	hypothetical protein	313	A	G	105/224	K	E	no domain impacted
rbsA	ribose import ATP-binding protein RbsA	7	C	T	3/534	H	Y	P-loop containing nucleoside triphosphate hydrolase
HP (7)	hypothetical protein	1789	T	A	597/633	L	I	non cytoplasmic domain
pgsA	CDP-diacylglycerol–glycerol-3-phosphate 3-phosphatidyltransferase	17	G	A	6/202	K	R	non cytoplasmic domain
lysS	lysine tRNA ligase	635	A	T	212/489	H	L	tRNA lysine domain protein
HP (8)	hypothetical protein	76	A	G	26/3325	T	A	transmembrane region
HP (9)	hypothetical protein	307	G	A	103/110	V	I	no domain impacted
pepA	cytosol aminopeptidase	566	A	T	189/453	E	V	modif proche des Zn binding sites
dnaK	chaperone protein dnaK interproscan	843	G	A	281/598	M	I	actin, chain A, domain 4
MIB	immunoglobulin-blocking virulence protein	1550	C	T	517/743	A	V	IgG blocking virulence domain (IPR030942)
		1852	C	T	618/743	H	Y	no domain impacted
gpmI	2,3-bisphosphoglycerate-independent phosphoglycerate mutase	551	A	G	184/498	Y	C	residu between active site (aa 182 and aa 188)
unk (3)	unknown DUF285 family protein	49	G	T	17/302	A	S	signal peptide
HP (10)	hypothetical protein	359	A	T	120/474	E	V	non cytoplasmic domain
unk (4)	unknown sugar ABC transporter permease	374	T	C	125/355	I	T	transmembrane region
yoaB	calcium-transporting ATPase 1	52	G	A	18/910	A	T	cation transporting P-type ATPase domain
HP (11)	hypothetical protein	217	A	G	73/97	K	E	no domain impacted
unk (5)	unknown glycerol ABC transporter substrate binding protein	530	C	T	177/618	A	V	non cytoplasmic domain
rluC	ribosomal large subunit pseudouridine synthase C	204	A	T	68/284	Q	H	RNA binding S4 domain / RNA binding motif
HP (12)	hypothetical protein	247	G	A	83/168	G	R	no domain impacted
rnc	ribonuclease III	336	A	T	112/229	A	T	RNAse III (4-130 aa) domain
